# Supplementary material for: Psychometric evaluation of Korean version of COVID-19 fear scale (K-FS-8): A population based cross-sectional study
Source: PLoS One. 2023 Mar 9;18(3):e0282589. doi: 10.1371/journal.pone.0282589 (PMC9997981; doi:10.1371/journal.pone.0282589)
Supplement: S1 File — (DOCX) [file pone.0282589.s001.docx]

**Supplementary file 1**. The Korean version of fear scale (K-FS-8)

1. 코로나19에 대한 생각은 나를 두렵게 한다
   (The thought of COVID-19 scares me)
2. 코로나19를 생각할 때 나는 긴장감을 느낀다
   (When I think about COVID-19, I feel nervous)
3. 코로나19를 생각할 때 나는 기분이 나빠진다
   (When I think about COVID-19, I get upset)
4. 코로나19를 생각할 때 나는 우울해 진다
   (When I think about COVID-19, I get depressed)
5. 코로나19를 생각할 때 나는 초조해 진다
   (When I think about COVID-19, I get jittery)
6. 코로나19를 생각할 때 나는 심장이 더 빨리 뛴다
   (When I think about COVID-19, my heart beats faster)
7. 코로나19를 생각할 때 나는 마음이 편치 않다
   (When I think about COVID-19, I feel uneasy)
8. 코로나19를 생각할 때 나는 불안감을 느낀다
   (When I think about COVID-19, I feel anxious)
