# Supplementary material for: Psychometric evaluation of Korean version of COVID-19 fear scale (K-FS-8): A population based cross-sectional study
Source: PLoS One. 2023 Mar 9;18(3):e0282589. doi: 10.1371/journal.pone.0282589 (PMC9997981; doi:10.1371/journal.pone.0282589)
Supplement: S2 File — (DOCX) [file pone.0282589.s002.docx]

**Supplementary file 2**. The additional IRT plots

***Scale information and conditional standard errors***

One especially useful IRT capability is the ability to accumulate information for separate elements to produce a scale information function. A scale information function summarises how well items give statistical information about the latent feature overall. Scale information values may also be used to construct conditional standard errors, which show how exactly scores can be assessed across different theta values.





**Supplementary figure 1. Scale information and conditional standard errors**

***Conditional reliability***

IRT approaches scale reliability differently from standard classical test theory methodologies that use coefficient alpha or omega. The CTT method assumes that reliability is determined by a single value that applies to all scale scores.





**Supplementary figure 2. Conditional reliability**

***Scale characteristic curve***

To calculate the ratings, we employed a latent trait scoring approach known as anticipated a posteriori (EAP) estimate. Once model-based theta score estimations are produced, it is common to want to convert those estimates back to the original scale measure. A scale characteristic function converts estimated theta scores to predicted true scores in the original scale measure. This conversion back to the original scale measure gives a more familiar framework for analysing results. Predicted true scores in this study relate to K-FS-8 metric scores (8 to 40) that are expected as a consequence of projected student theta scores.





**Supplementary figure 3. Scale characteristic curve**
