# Supplementary figures and images for: Psychometric evaluation of Korean version of COVID-19 fear scale (K-FS-8): A population based cross-sectional study
Source: PLoS One. 2023 Mar 9;18(3):e0282589. doi: 10.1371/journal.pone.0282589 (PMC9997981; doi:10.1371/journal.pone.0282589)

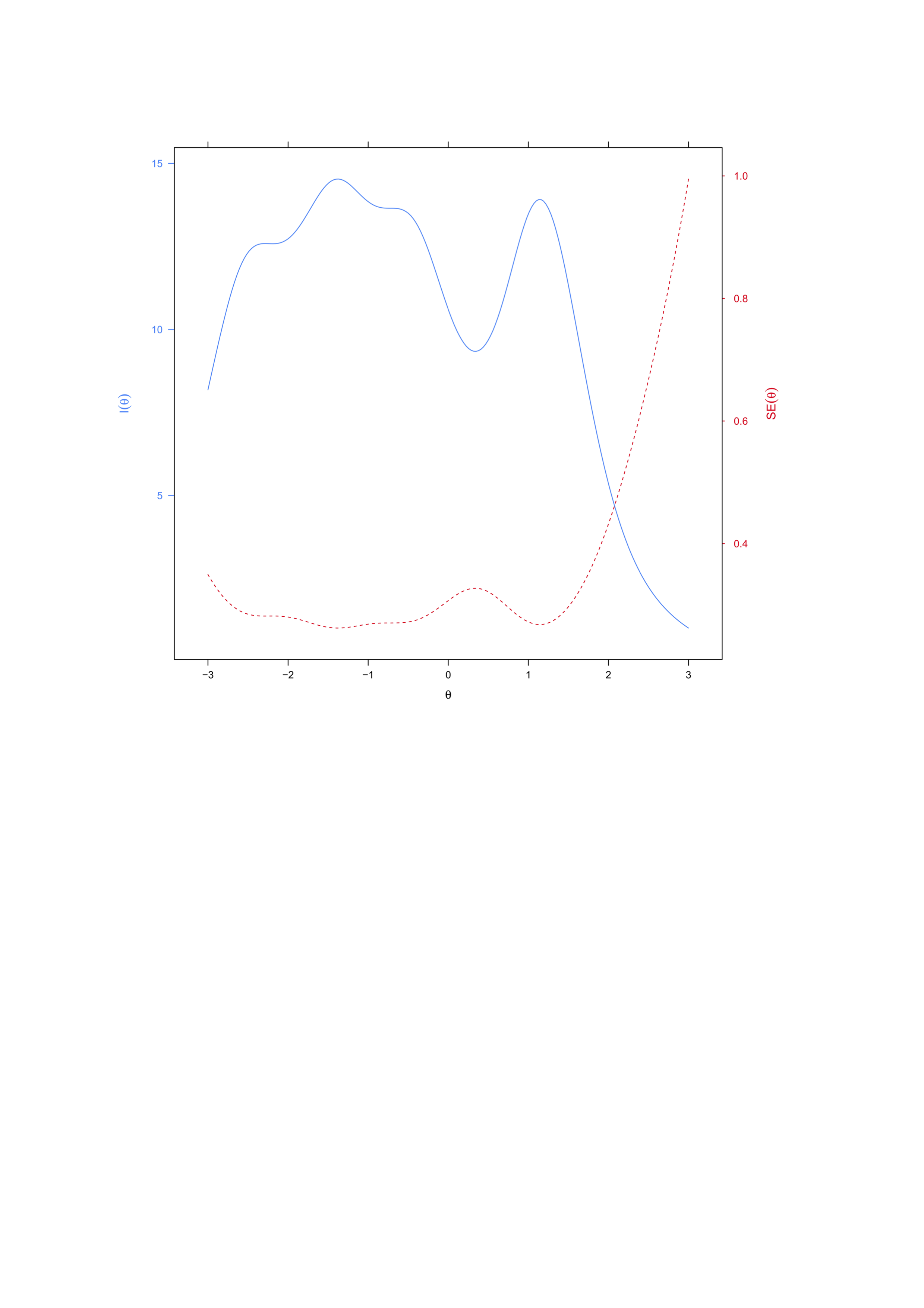

Supplement: S1 Fig — (TIFF) [file pone.0282589.s004.tiff]

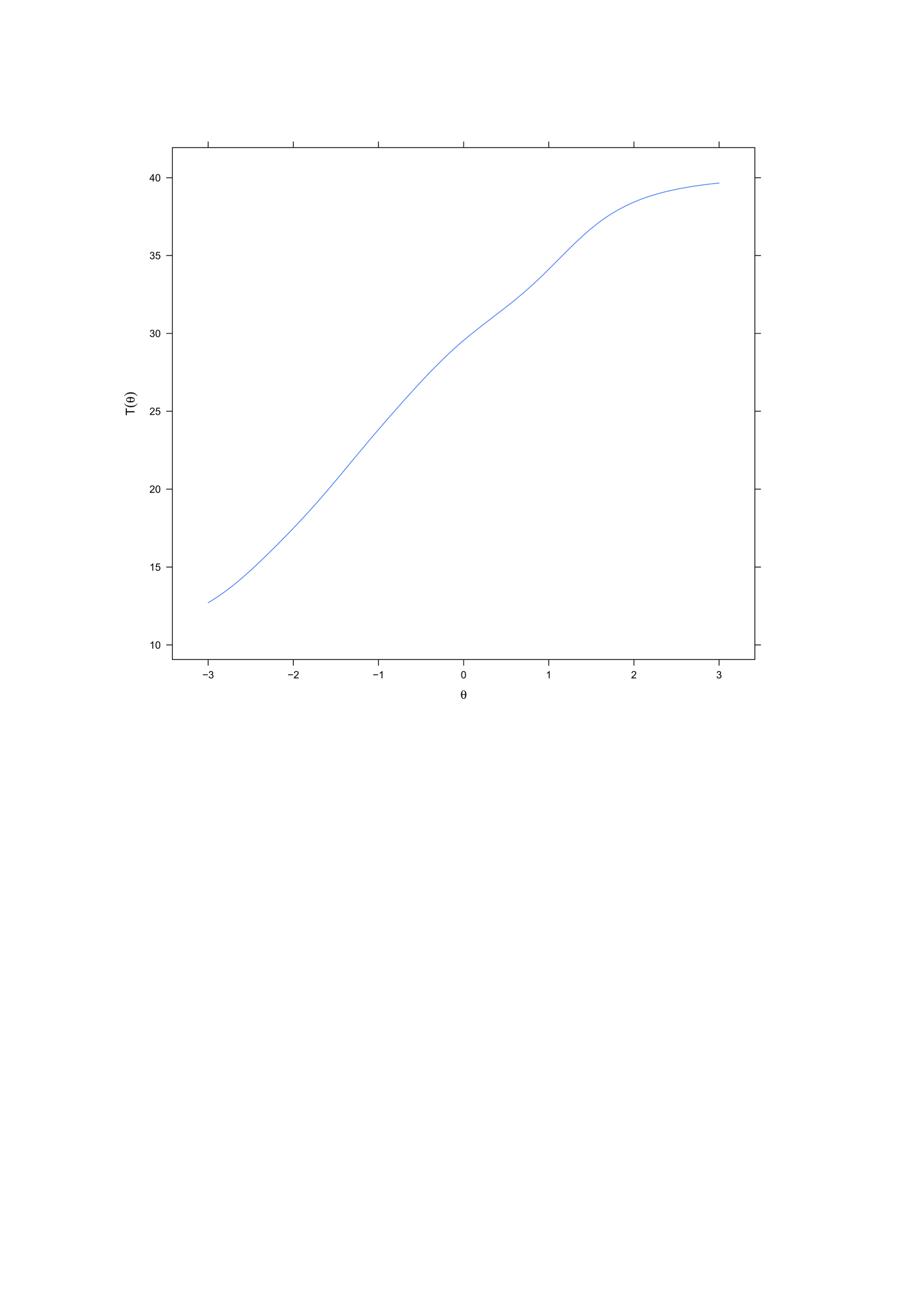

Supplement: S2 Fig — (TIFF) [file pone.0282589.s005.tiff]
