# Supplementary material for: Psychometric evaluation of Korean version of COVID-19 fear scale (K-FS-8): A population based cross-sectional study
Source: PLoS One. 2023 Mar 9;18(3):e0282589. doi: 10.1371/journal.pone.0282589 (PMC9997981; doi:10.1371/journal.pone.0282589)
Supplement: Attachment — Submitted filename: PONE-D-22-30798_comments.pdf [file pone.0282589.s007.pdf]

of depression, and 22.6% had the risk of anxiety[20]. However, with respect to the fear of COVID-19, South Korea has not yet been evaluated from a large sample owing to a lack of validated scales for measuring fear during the pandemic of infectious diseases including COVID-19. Therefore, this study aimed to conduct a psychometric evaluation of the Korean

adverse health effects. Healthcare professionals can use the K-FS-8 to screen fear of the COVID-19 and related major public health crises with effectiveness and efficiency among their clients and the general public in community and primary care settings to achieve early recognition of clinical deterioration in psychological well-being and health behaviours.

1. Please discuss existing scales (or related scales) in the native social context, and outline their shortcomings to further establish the significance of this study, it is recommended to provide stronger justification to use a scale from a different sphere (breast cancer). Furthermore, it is not clear how this scale will benefit in applied settings. Is it intended as a diagnostic measure? In the conclusion it was mentioned that healthcare professionals should use the K-FS-8 as a screening tool, can you elaborate on this point or provide examples from other countries where fear scales were used effectively in clinical settings to achieve favorable outcomes?

been widely cited in the literature. The BCFS was developed on the robust theoretical framework—Extended Parallel Process Model (EPPM)[22] and Health Belief Model (HBM)[23], which establishes the definitions of fear, threat and barrier based on a profound understanding of their nature[21]. Even though the fear scale was initially developed to measure the fear of breast cancer, the scale was validated for measuring the fear of COVID-19 in mainland China and Hong Kong[24].

This study has several limitations. The fear scale(i.e., BCFS) was initially designed for the fear of breast cancer[21]. However, BCFS was developed on the basis of a theoretical framework—the combination of EPPM and HBM—which have been widely used for healthcare practice and research and hence can be applied for other diseases and/or populations. As this study only recruited adults, further validation in younger population

2. Breast cancer and COVID-19 are two fundamentally different health hazards, which may raise concerns if the same instrument is used to measure fear towards each. For example, breast cancer involves a hereditary risk that may not be shared by a pandemic disease. Similarly, a pandemic disease like COVID-19 spreads through contagion while breast cancer does not. Therefore, the fear towards each may manifest differently. It is not clear if the shared theoretical framework (EPPM/HBM) involve an overlap between non-communicable diseases and communicable diseases.

   These points do not undermine the current study, but the authors are strongly recommended to elaborate on their justification. It is recommended to get in touch

with the original authors of the BFCS, and discuss these theoretical foundations that may prevent or facilitate the use of BFCS towards a communicable health hazard like COVID-19. Providing a citation to the correspondence with the BFCS authors' approval will bolster this study's theoretical foundations, especially considering that only one word was changed to convert the BFCS into a COVID fear scale.

> highlights the necessity for delivering psychological interventions that alleviate fear to the Korean population. Such interventions will enhance their psychological well-being and prevent complications caused by the fear.

3. Please provide examples from the literature on what kind of interventions are feasible in this situation.
