# Supplementary material for: Psychometric evaluation of Korean version of COVID-19 fear scale (K-FS-8): A population based cross-sectional study
Source: PLoS One. 2023 Mar 9;18(3):e0282589. doi: 10.1371/journal.pone.0282589 (PMC9997981; doi:10.1371/journal.pone.0282589)
Supplement: Attachment — Submitted filename: Reviewer comments.docx [file pone.0282589.s006.docx]

December 19, 2022

Thank you for the opportunity to review the paper “Psychometric evaluation of Korean version of COVID-19 fear scale (K-FS-8): a population based cross sectional study”, which I thoroughly enjoyed. The authors attempt to translate and validate the COVID-19 fear scale in Korean population by adapting a fear scale designed originally for breast cancer focusing on multiple aspects of validity.

By validating the scale, the authors make a valuable addition to scales existent in mental health research, in context of Korea. However, I believe the manuscript would benefit by considering the following points. Please consider the comments as constructive and also note that I do not have expertise in psychometrics.

Abstract

Please consider not using acronyms in the abstract. Save it later for the manuscript where the terms are first used.

Introduction

I believe that the introduction would benefit from adding on to the literature on existence of other scales for measuring fear during covid- 19, commonly in use globally and in context of Korea. A quick search yielded many interesting publications with covid- 19 fear scale, the most widely used and it appears that this has been validated in Korea as well in 2021.

Please refer to the following few articles for the reference:

[The Korean Version of Fear of COVID-19 Scale: Psychometric Validation in the Korean Population - PubMed (nih.gov)](https://pubmed.ncbi.nlm.nih.gov/33849243/)

[Instruments to measure fear of COVID-19: a diagnostic systematic review | BMC Medical Research Methodology | Full Text (biomedcentral.com)](https://bmcmedresmethodol.biomedcentral.com/articles/10.1186/s12874-021-01262-5)

Further, the introduction can be shortened in their first and third paragraphs.

The authors mention no validated fear scales for COVID-19 exists in Korea as the major rationale for the conduct of the study, though they have included this in the discussion section. Please justify the statement on what the authors meant.

Also, I suggest the authors be explicit in why they wanted to validate the scale that was originally intended for breast cancer. Is there any theory to guide?

Materials and Methods

The authors mention that face validity and cognitive interviewing were done in phase 1. Do the authors have any data to demonstrate the content validity and what changes were made in the original translation after these steps? How were the findings analyzed? It would be helpful if the authors provide a table to the demographics on whom cognitive interviewing was done for validity of the findings.

I understand this was a survey, but based on research question, did the authors have any estimation for sample size prior to conducting the study?

The authors state that PHQ-4 and PC-PTSD-5 were adopted for this study to assess convergent validity. What do the authors mean with this statement? Were these instruments not already validated in the Korean population?

For known- group validity, T test and ANOVA have been used. I wonder if the authors checked for the distribution of the data before going with the tests. Please clarify.

Item response theory (IRT)- were the assumptions for IRT met?

Results

Were there any missing data on any items? Please clarify.

Discussion

The discussion would benefit by including more literature in relation to fear scales in the local and the global context and the limitations with the existing scales.

The authors mention that fear might be higher in Korean sample. What do the authors mean?

Also, the fact that fear is a natural response to any threat, perceived or real and is protective. So, high level of fear during covid-19 at a time when nothing was known about it is justified. I wonder why the authors focus on the statement that high level of fear means it always needs intervention. As also reported in the conclusion. I believe the discussion should focus more on clinical or morbid fear that necessitates intervention, by doing a more through investigation. I suggest the authors consider this in discussion as well as conclusion.

The authors have pointed out the limitations with the study, which is good.

A critical aspect is that even if the correlations are high and indicate reliability, they might not be a valid indicator of what we intend to measure. So, this is also a limitation to studies which focus on one aspect of validity. The authors can consider this point also to highlight in their discussion. Other aspects such as divergent validity could also be discussed.

I wish the authors all the best.
